# 肿瘤间质比是非小细胞肺癌独立的预后因素

**DOI:** 10.3779/j.issn.1009-3419.2013.04.04

**Published:** 2013-04-20

**Authors:** 兆峰 王, 红兵 刘, 仁德 赵, 鹤 张, 春花 刘, 勇 宋

**Affiliations:** 1 210002 南京, 南京军区南京总医院呼吸内科, 南京大学医学院临床学院 Department of Respiratory Medicine, Nanjing General Hospital of Nanjing Command, Clinical School of the Medical College of Nanjing University, Nanjing 210002, China; 2 271200 新泰, 新泰市人民医院肿瘤科 Department of Oncology, Xintai Municipal People's Hosital of Shandong Province, Xintai 271200, China

**Keywords:** TSR, 肺肿瘤, 预后, TSR, Lung neoplasms, Prognosis

## Abstract

**背景与目的:**

已有研究表明, 作为一种病理形态定量方法, 肿瘤间质比(tumor-stroma ratio, TSR)在一些实体瘤中已经被证实是一种新的、可靠的预后因子。本研究旨在探讨TSR在Ⅰ期-Ⅲ期术后非小细胞肺癌(non-small cell lung cancer, NSCLC)的预后价值。

**方法:**

对73例Ⅰ期-Ⅲ期NSCLC外科手术切除标本的蜡块进行切片, 运用苏木素染色法染色。在低倍镜镜下选取肿瘤组织内间质浸润最明显的区域, 高倍镜下计数该区域单视野内肿瘤细胞及肿瘤内间质分别占该视野的百分比(即TSR), 根据TSR将病例分为两组:肿瘤组织内肿瘤细胞面积 < 50%视野定义为间质丰富组(stroma-rich group), 而肿瘤细胞面积≥50%视野定义为间质稀少组(stroma-poor group)。随访两组患者的总生存期(overall survival, OS)。采用SPSS 16.0软件进行统计处理实验数据, 采用卡方检验分析间质稀少组及间质丰富组与不同临床特征之间的关系, 用*Kaplan-Meier*生存分析和*Cox*回归分析等统计方法分析两组间预后的差异。

**结果:**

在73例组织标本中, 间质丰富组有27例, 间质稀少组有46例, 所占比例分别为37%、63%。卡方检验分析此两组之间在性别、年龄吸、烟史、肿瘤大小、病理分型及pTNM分期等病理特征间无统计差异; *Kaplan-Meier*生存分析显示, TSR与NSCLC患者的OS明显相关(*Log-rank* test, *P*=0.014), 间质丰富组的患者OS明显低于间质稀少组的患者。多因素*Cox*、回归分析显示TSR和pTNM是影响NSCLC独立的预后因素(HR=1.832, 95%CI:1.017-3.299;HR=1.953, 95%CI:1.284-2.970)。

**结论:**

TSR是NSCLC独立的预后因素。

## Abstract (en)

**Background and objective:**

The different expression of tumor-stroma ratio (TSR) have been proved to be a new and reliable independent prognostic factor in some solid tumors.The aim of the study is to test the different expression of TSR and its prognostic significance in non-small cell lung cancer (NSCLC).

**Methods:**

A total of 73 patients who underwent surgery resection for NSCLC were included in this study.TSR was assessed visually on the hematoxylin-stained tissue sections of surgical specimens.Patients with more than 50% intratumor stroma were quantified as the stroma-rich group and those with less than 50% as the stroma-poor group.

**Results:**

In 73 cases of tissue samples, 46 cases were included in the stroma-poor group, while 27 cases in stroma-rich group.The different expression of TSR in NSCLC tissue was not correlated with gender, age and pathological type, lymph node metastasis, tumor size, pTNM staging, and so on.*Kaplan-Meier* survival analysis showed that the different expression of TSR was significantly correlated with survival days (*P*=0.014).*Cox* regression analysis showed that not only different expression of TSR is a independent prognostic factor for NSCLC (HR=1.832, 95%CI:1.017-3.299), but also pTNM staging (HR=1.953, 95%CI:1.284-2.970).

**Conclusion:**

The different expression of TSR might be an independent prognostic factor in NSCLC.

近期发表的文献^[1]^显示:2012年肺癌的死亡率仍将处于各种肿瘤首位, 新发病例居第二位。在诸多西方国家, 近15年肺癌的发病率已达高峰并呈下降趋势, 而我国肺癌的发病率却呈持续上升趋势^[2]^。鉴于肺癌高发病率和死亡率, 提高肺癌的诊治水平, 改善患者预后已成为亟待解决的难题。近年来, 非小细胞肺癌(non-small cell lung cancer, NSCLC)作为最常见的组织病理类型研究较多。生存相关的研究^[3-6]^已证实年龄、KPS评分、TNM分期等是NSCLC的独立预后因素。但是, 我们是否可以在进行病理诊断的同时根据组织标本的病理形态表现预测患者的预后呢？Mesker、Wang等^[7-11]^研究表明:在结直肠癌、乳腺癌、食管鳞癌及食管腺癌等多种实体瘤内, 肿瘤组织内肿瘤与间质的比例(tumor-stroma ratio, TSR)被证明是一种新的、可靠的独立预后因子, 且此评估方法简单, 切实可行, 重复性好。但TSR对NSCLC患者的预后价值目前尚无相关研究, 本实验旨在证实TSR在NSCLC中的预后价值。

## 1 材料与方法

### 1.1 材料

选取南京军区南京总医院2000年1月-2007年1月手术切除的经常规病理证实的NSCLC标本73例。73例NSCLC中, 男性50例, 女性23例; 年龄30岁-75岁, 中位年龄59岁。所有NSCLC病例术前未经化疗和放疗, 术后病理证实为NSCLC(鳞癌35例, 腺癌33例, 腺鳞癌5例), 术后经过2个-4个疗程的以铂类为基础的化疗。按照UICC分期委员会第7版TNM分期标准, 分为Ⅰ期22例, Ⅱ期28例, Ⅲ期23例。

### 1.2 方法

将术后组织常规石蜡包埋, 制片成厚度为4 µm切片。采用苏木素染色法, 按常规程序脱蜡和水化。PBS液冲洗后, 用苏木素(北京中杉金桥公司)进行染色。梯度酒精脱水, 二甲苯透明, 中性树胶封片。

### 1.3 实验结果的判定

实验结果根据参考文献^[7]^判定:常规镜检苏木素染色后的切片, 50×放大倍数下选取肿瘤浸润最明显的区域。常规认为该区域有两部分组成:肿瘤细胞部分和间质部分, 用百分比表示。然后, 在该区域内100×倍放大倍数下评估单视野范围内肿瘤细胞面积所占该视野的百分比(carcinoma percentage, CP), 剩余百分比即为间质百分比(例:如果肿瘤细胞面积占该视野40%, 间质成分面积所占百分比即为60%。取10%为取值区间, 即:20%、30%等), 计算肿瘤内部的TSR。选取2个-4个视野进行评估, 取CP值最低的一个确定为最终取值。因计算的为肿瘤内部的TSR, 故该视野范围内间质周围必须是有肿瘤细胞浸润。不能选取间质较多, 间质周围无肿瘤细胞的区域进行评估。根据TSR评估结果, 选取CP为50%的cut-off值, 将病例分为两组:将CP值< 50%病例定义为间质丰富组(stroma-rich group)而CP值≥50%视野定义为间质稀少组(stroma-poor group)。以上评估过程主要由2名病理专家完成, 当2名专家意见不一致时根据第32名病理专家的意见确认最终结果。

### 1.4 随访

患者从手术日期开始随访, 至患者死亡结束, 生存者至2012年5月31日终止随访。

### 1.5 统计处理

采用SPSS 16.0软件进行统计处理, 采用卡方检验、*Kaplan-Meier*生存分析和*Cox*回归分析。*P* < 0.05为差异有统计学意义。

## 2 结果

### 2.1 TSR在NSCLC组织中的分布

根据以上试验方法, 在73例NSCLC组织中, 间质丰富组27例, 间质稀少组46例, 比例分别为37%、63%。不同TSR在肺鳞癌及腺癌中病理形态学示例如图 1所示。

**1:**
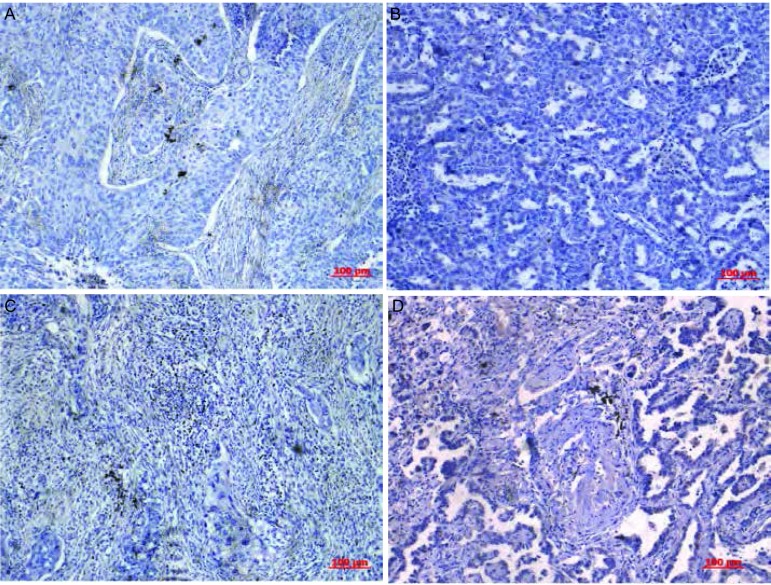
TSR在不同病理类型肺组织中的表达。A:鳞癌间质稀少组; B:腺癌间质稀少组; C:鳞癌间质丰富组; D:腺癌间质丰富组(×100)。 The expression of TSR in lung tissue of different kinds of histology.A:The stroma-poor group of squamous cell lung carcinoma; B:The stroma-poor group of lung adenocarcinoma; C:The stroma-rich group of squamous cell lung carcinoma; D:The stroma-poor group of lung adenocarcinoma (×100).

### 2.2 TSR分组与临床病理参数的关系

根据TSR结果, 间质稀少组与间质丰富组在性别、年龄、吸烟、肿瘤大小、病理类型及淋巴结转移及pTNM分期间无统计学差异。结果见表 1。

**1:** TSR与不同临床病理特征间的关系 The relationship between TSR and different clinical and pathological features

Characteristics	Total		Stroma-poor group		Stroma-rich group		*P*
	*n*	%	*n*	%	*n*	%	
Gender							
Men	50	68.5	35	76.1	15	55.6	0.068
Women	23	31.5	11	23.9	12	44.4	
Age							
< 60	34	46.6	22	47.8	12	44.4	0.780
≥60	39	53.4	24	52.2	15	55.6	
Smoking history							
< 20 P.Y	39	53.4	26	56.5	13	48.1	0.489
≥20 P.Y	34	46.6	20	43.5	14	51.9	
Tumor diameter							
< 4 cm	26	35.6	16	34.8	10	37.0	0.846
≥4 cm	47	64.4	30	65.2	17	63.0	
Histology							
Adeno CA	33	45.2	24	52.2	9	33.3	0.287
SCC	35	47.9	19	41.3	16	59.3	
Adeno-SC CA	5	6.90	3	6.50	2	7.40	
Lymphatic metastasis							
NO	33	45.2	23	50.0	10	37.0	0.283
Yes	40	54.8	23	50.0	17	63.0	
pTNM stage							
Ⅰ	22	30.1	15	32.6	7	25.9	0.429
Ⅱ	28	38.4	19	41.3	9	33.3	
Ⅲ	23	31.5	12	26.1	11	40.8	
Differentiation grade							
Well	18	24.7	10	21.7	8	29.6	0.325
Moderate	34	46.5	20	43.5	14	51.9	
Poor	21	28.8	16	34.8	5	18.5	
P.Y:package.year; Adeno CA:adenocarcinoma; SCC:squamous carcinoma; AdCA:adenosquamous carcinoma. *P* < 0.05 was considered significant.							

### 2.3 不同TSR分组与NSCLC预后的关系

73例NSCLC患者从手术日期开始随访, 至患者死亡结束, 未死亡的至2012年5月31日截止随访。用*Kaplan-Meier*生存分析和*Log-rank*检验统计分析, TSR两分组之间OS差异有统计学意义(*P*=0.014), 结果显示间质稀少组患者中位生存时间为1, 169 d, 间质丰富组的患者中位生存时间为808 d。间质丰富组的患者OS明显低于间质稀少组的患者(图 2)。对性别、年龄、吸烟、肿瘤大小、病理类型、淋巴结转移、pTNM分期、分化程度及TSR分组9个因素进行Cox模型多因素分析(表 2)可见, TSR是影响NSCLC预后的独立因素(HR=1.832, 95%CI:1.017-3.299), pTNM分期也是影响NSCLC独立预后因素(HR=1.953, 95%CI:1.284-2.970), 而性别、年龄、吸烟史、肿瘤大小、病理类型、淋巴结转移及分化程度均非NSCLC预后的独立因素。

**2:**
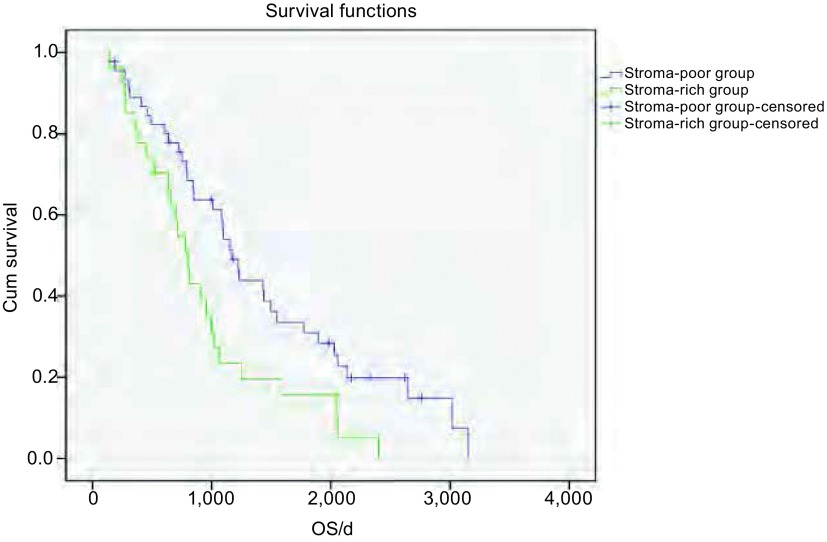
73例NSCLC患者*Kaplan-Meier*生存分析。结果显示间质稀少组患者中位生存时间为1, 169 d, 间质丰富组的患者中位生存时间为808 d。间质丰富组的患者OS明显低于间质稀少组的患者(*P*=0.014)。 *Kaplan-Meier* survival analysis of 73 cases with NSCLC.Results show that the median survival time in the stroma-poor group was 1, 169 d, and 808 d in the stroma-rich group.OS days of patients in stroma-rich group were significantly lower than the stroma-poor group (*P*=0.014).OS:Overall survival.

**2:** NSCLC预后因素多变量*Cox*回归分析 Multivariate *Cox* regression analysis of prognostic factors in NSCLC

Variable	*P*	HR	95.0%CI for HR	
			Lower	Upper
Gender	0.770	0.907	0.470	1.751
Age	0.300	0.725	0.395	1.332
Smoking history	0.394	1.330	0.690	2.562
Tumor diameter	0.271	0.731	0.418	1.278
Histology	0.077	0.658	0.414	1.047
Lymphatic metastasis	0.310	1.355	0.754	2.437
pTNM stage	0.012	1.953	1.284	2.970
Differentiation grade	0.788	1.059	0.696	1.613
TSR groups	0.044	1.832	1.017	3.299

## 3 讨论

本实验采用苏木素法对73例NSCLC手术切除标本的TSR进行检测, 根据TSR评估结果, 选取CP%为50%的cut-off值, 将病例分为两组:间质丰富组(stroma-rich group)和间质稀少组(stroma-poor group)。统计分析显示:间质丰富组患者预后明显差于间质稀少组, 两组OS差异有统计学意义(*P*=0.014), 并证明TSR是NSCLC独立的预后因子(HR=1.832, 95%CI:1.017-3.299)。该实验对TSR在NSCLC中的预后价值做出了评价。

目前, 关于TSR在实体瘤中的预后价值, 研究尚少。荷兰学者Mesker等在前期的研究中发现:在实体瘤内, 与肿瘤组织接触的间质组织可能影响患者的预后。为进一步证实上述现象, Mesker等^[7]^用HE染色的方法对122例Ⅰ期-Ⅲ期的结直肠癌患者手术组织进行病理形态分析及评估, 并随访患者预后。研究结果发现:选取CP为50%的cut-off值对患者进行分组, CP≥50%组的患者OS和无病生存(disease-free survival, DFS)均长于CP < 50%组。该实验同时发现, 随着结直肠癌分期的递进, CP值越小。该实验结果与本实验相似, 但两实验结果统计分析未见TNM分期与TSR有相关性, 由于两实验样本量相对较少, 需要增加样本量以进一步研究。为进一步研究TSR在结直肠癌中的价值, Mesker等^[10]^开展了另一组含135例Ⅰ期-Ⅱ期结直肠癌患者的研究, 预后分析进一步证实了TSR在结直肠癌中的预后价值。Courrech Staal等^[9]^对93例食管腺癌术后组织的TSR进行评估并随访患者预后, 发现CP高组患者的DFS明显长于CP低组, 并证实TSR是食管腺癌独立的预后因素(HR=2.006, 95%CI:1.181-3.407)。关于TSR与NSCLC预后的关系, 目前尚无相关报道。本实验结果显示:通过对73例NSCLC术后组织切片苏木素染色后发现, 间质稀少组患者中位生存时间为1, 169 d, 间质丰富组的患者中位生存时间为808 d。间质丰富组的患者OS明显低于间质稀少组的患者, 多因素*Cox*回归分析显示TSR是影响NSCLC预后的独立因素(HR=1.832, 95%CI:1.017-3.299), 与Mesker等以上研究结果相似。本实验同时证实TNM分期是NSCLC独立的预后因素, 这与以往研究结论一致。

肿瘤组织是由肿瘤细胞和复杂的间质组成。在正常组织内间质具有屏障的作用, 但在肿瘤组织内, 作为肿瘤微环境的间质成分却对肿瘤的浸润转移有易化作用^[12]^。新近研究^[13, 14]^表明, 在肿瘤演进过程中, 上皮间质转化(epithelial-mesenchymal transition, EMT)是一个重要的决定性过程, 此过程通过多个细胞内通路参与肿瘤细胞侵袭转移乃至“干细胞性”转变等病理过程。在癌变的早期, 肿瘤细胞的增殖未突破基底膜, 与周围间质隔开, 此为原位癌。当原位癌发展成浸润性癌时, 癌细胞穿过基底膜侵犯间质, 诱导正常间质转化为“活性间质”^[15]^, 此过程促进了成纤维细胞的增殖及细胞外基质(extracellularmatrixc, ECM)的沉积^[16]^, 从而扩充了肿瘤间质。而作为间质重要成分的成纤维细胞, 不仅在创伤愈合等多种炎症反应中起作用, 而且是肿瘤的关键性特征^[17]^。近年来, 关于一种肿瘤间质内特殊的成纤维细胞-肿瘤相关成纤维细胞(cancer-associated fibroblasts, CAFs)因在肿瘤的发生发展中的特殊作用而逐渐得到重视。大部分侵袭性肿瘤的间质都会有CAFs, CAFs具有较强的收缩能力, 它可以合成ECM, 从而刺激上皮细胞的增值, 通过旁分泌不同生长因子及细胞因子, 如:肝细胞生长因子(hepatocyte growth factor, HGF)、胰岛素生长因子(insulin-like growth factors, IGF)、神经生长因子(neuron growth factor, NGF)等, 活化后的CAFs可分泌高水平的细胞外金属基质蛋白酶, 从而促进ECM组分的改变, 影响上皮肿瘤细胞的演进^[18-20]^。除了对原发灶肿瘤细胞的作用, Olaso等^[21]^研究发现在转移灶内活化的CAFs同样可以促进肿瘤的增值。Balsamo等^[22]^发现CAFs还能够调节免疫细胞功能以使肿瘤细胞获得免疫逃逸。由以上研究可见, 肿瘤组织内的间质成分通过多种通路参与了肿瘤发生、增殖及浸润转移的过程, 从而影响患者的预后。但CAFs与其它间质成分之间如何相互影响, 以及EMT与CAFs之间的确切关系, 这些相互作用机制与肿瘤的发生发展之间的确切关系, 目前尚未完全清楚。

尽管本实验对TSR在NSCLC中的预后价值做出了评价, 但本实验也存在一些缺陷, 如:本实验主要研究了NSCLC中常见的鳞癌、腺癌及腺鳞癌等病理类型, 但NSCLC分型复杂, 仅腺癌就有多种不同分类, 形态学差异较大, 因此有待于扩大样本量, 完善病理类型, 进一步确认实验结果。

总之, 本研究提示通过组织病理形态分析有可能为NSCLC患者预后提供可靠信息, 且该方法简单可行。本实验同时证实TSR是NSCLC患者独立的预后因素。但是, 关于TSR与NSCLC之间的机制关系尚未完全明确, 尚待进一步研究。
